# The Impact of Hand Strength on HbA1c, Body Mass Index and Body Composition by Group According to Sedentary Behaviour: Cross-Sectional Study in Japanese Patients with Type 2 Diabetes Mellitus

**DOI:** 10.21315/mjms2024.31.3.14

**Published:** 2024-06-27

**Authors:** Shuhei Nakanishi, Masashi Shimoda, Tomohiko Kimura, Yukino Katakura, Junpei Sanada, Yoshiro Fushimi, Yuichiro Iwamoto, Hideyuki Iwamoto, Tomoatsu Mune, Kohei Kaku, Hideaki Kaneto

**Affiliations:** Division of Diabetes, Metabolism and Endocrinology, Kawasaki Medical School, Okayama, Japan

**Keywords:** diabetes mellitus type 2, sedentary behaviour, hand strength, glycated haemoglobin, body composition

## Abstract

**Background:**

The impact of hand strength in consideration of sedentary behaviour on diabetes management in patients with type 2 diabetes mellitus (T2DM) is unclear. The purpose of this study was to examine the impact of hand strength on HbA1c, body mass index (BMI) and body composition by group according to the duration of sedentary behaviour in Japanese patients with T2DM.

**Methods:**

In this retrospective, cross-sectional, single-centre study, hand strength standardised by bodyweight (GS) and sedentary time (ST), were obtained and analysed in a total of 270 Japanese T2DM outpatients in 2021. After dividing the patients into four categories of median values (high and low GS, and long and short ST), odds ratios (ORs) for good control of HbA1c, BMI, waist circumference (WC) and intra-abdominal fat (IAF) were investigated using logistic regression models.

**Results:**

The high GS/short ST group was found to have a significantly higher (OR = 2.01; 95% CI: 1.00, 4.03; *P* = 0.049) for controlled HbA1c compared with that of the low GS/long ST group. The high GS/short ST and the high GS/long ST groups had significantly higher ORs for controlled BMI, WC and IAF compared with the OR of the low GS/long ST group. In addition, the ORs were significantly increased with a positive trend in order from low GS/long ST, low GS/short ST, high GS/long ST, to high GS/short ST in all models (*P* < 0.001 for trend).

**Conclusion:**

Hand strength, with modest effects from sedentary behaviour, could be helpful for diabetes management in T2DM patients.

## Introduction

The goal of type 2 diabetes mellitus (T2DM) management is to extend healthy life by avoiding diabetic micro and macrovascular complications and to improve quality of life equivalent to that of their counterparts without diabetes, particularly given that T2DM is now reaching ‘pandemic’ proportions and threating worldwide health and economic growth due to complications from the disease (1–3). In 2021, there were about 529 million people living with diabetes worldwide, and the global age-standardised total diabetes prevalence was around 6.1% (3). Important means for attaining this objective are the control of blood glucose levels, bodyweight and body composition (4).

Hand strength, a simple and objective measure, might be useful as a surrogate for overall muscular strength because it is highly correlated with other muscular strength measures, including elbow flexion, knee extension, trunk flexion and trunk extension (5–8). Moreover, muscular strength might be associated with skeletal muscle mass, a significant site of glucose disposal and insulin action (9). Thus, hand strength as a surrogate for muscular strength could be considered a marker for the management of risks associated with T2DM (10). Weakness in muscular strength might also indicate decreased physical activity, which affects glycaemic control, bodyweight and body composition. Accordingly, hand strength might also be associated with glycaemic control in T2DM patients.

Meanwhile, an increasing number of studies have associated a longer duration of sedentary time (ST) with unfavourable metabolic and cardiovascular risk markers, independent of moderate or vigorous levels of physical activity (11, 12). Patients with T2DM are encouraged to decrease the duration of time spent daily in sedentary behaviour because extended ST is a known risk factor for poorer glycaemic control (13, 14). Such recommendations for avoiding sedentary behaviour raise the clinical question about whether the impact of hand strength is affected by the degree of sedentary behaviour in terms of treatment of T2DM. However, physicians might try to achieve glycaemic control by prescribing medication independent of any consideration of hand strength and sedentary behaviour. Accordingly, the impact of hand strength in consideration of sedentary behaviour on diabetes management in patients with T2DM is unclear. In this single-centre, cross-sectional study, we examined whether ST affected the impact of hand strength on HbA1c, body mass index (BMI), waist circumference (WC) and intra-abdominal fat (IAF) among outpatients with T2DM, based on the hypothesis that sedentary behaviour affected the impact of hand strength on metabolic factors such as HbA1c, bodyweight and body composition. Clarifying the relationship between hand strength and sedentary behaviour would affect exercise therapy for T2DM.

## Methods

### Study Participants and Patient Preparation

In this retrospective, cross-sectional, single-centre study, a total of 315 patients with T2DM among 2,475 patients who regularly visited the diabetes outpatient clinic at Kawasaki Medical School Hospital during the period from July 2021 to December 2021. The patients who underwent all measurements of hand strength, WC, IAF and ST information were considered eligible to participate and were included in this study. ST information was obtained for all patients based on Japan’s version of the International Physical Activity Questionnaire (IPAQ) short form (15, 16). Excluded from the study were patients with active retinopathy, end-stage renal disease, severe neuropathy, steroid use, younger than 20 years of age, older than 80 years old, who had difficulties in carrying out physical activity due to orthopaedic and other impairments such as fatigue or infections, low BMI of less than 18.5 kg/m^2^, high BMI of greater than 40 kg/m^2^ and those otherwise deemed inappropriate for the questionnaire by the attending physician. The final study sample consisted of 270 participants.

Hand strength was measured in both left and right hands with participants in a standing position using a dynamometer in units of kilograms (TKK 5401, Takei Scientific Instruments, Japan). Participants held the dynamometer at thigh level and were encouraged to exert the strongest possible force in each of their two hands. The mean hand strength calculated on the basis of measurements for both hands was used in the present analysis. WC was measured at the umbilical level in the late expiration phase with participants standing. IAF around the waist was estimated by bioelectrical impedance analysis (BIA) (Panasonic EW-FA90, Shiga, Japan), as reported previously (17). In brief, voltage at the umbilicus position was correlated significantly with IAF and was not affected much, if at all, by subcutaneous fat, which suggested that IAF could be calculated accurately based on voltage feedback. The correlation of BIA with the computed tomography (CT) measurement results was 0.88 (18). BMI was calculated by dividing weight in kilograms by height in meters squared.

The effect of hand strength on HbA1c level, BMI, WC and IAF was cross-sectionally investigated in the clinical setting described above, in consideration of ST, with the aim of clarifying impact of muscular strength and sedentary behaviour on T2DM treatment. The hospital’s ethics committee approved the study protocol and information pertaining to the study was provided to the public via the internet, instead of informed consent being obtained from each individual patient (No. 5245-00).

### Statistical Analysis

Categorical variables were expressed as numerals and percentages. Continuous variables were expressed as mean and standard deviation. Pearson’s chi-square tests were used for testing associations between categorical variables. Residual analyses were used to identify the specific cells making the greatest contribution to the chi-square test results. Each of the hand strength (kg) values was divided by participant bodyweight (kg) (GS), because hand strength was thought to be positively affected by bodyweight (19, 20). Given that the data regarding ST, GS, HbA1c, BMI, WC and IAF were not normally distributed, analyses were performed after logarithmic transformation. Continuous variables were analysed using analysis of covariance (ANCOVA) for comparisons with categorical variables. After multivariate tests, to determine if there were significant differences, Tukey’s tests were performed for post-hoc analysis. To precisely understand the impact of GS and ST on HbA1c, BMI, WC and IAF, the participants were divided into four groups by median GS by gender (0.47 in male, 0.34 in female) and ST (360 min/day): low GS/long ST (LL), low GS/short ST (LS), high GS/long ST (HL) and high GS/short ST (HS). In addition, to clarify the significance of GS as a determinant in controlled HbA1c, BMI, WC or IAF, with controlled or uncontrolled as the dependent variable (1 = controlled; 0 = uncontrolled), the odds ratios (ORs) of the LS, HL and HS groups in comparison with the LL group were estimated using logistic regression models. ‘Controlled’ HbA1c (*n* = 130) was defined as being lower than 7%, the target recommended by the Japan Diabetes Society (21). ‘Controlled’ BMI (*n* = 161) was defined as being lower than 25 kg/m^2^ among women and 27 kg/m^2^ among men, values higher than those reported to elevate mortality risk in a Japanese cohort (22). WC and IAF were defined as ‘controlled’ in accordance with the definition of metabolic syndrome in Japan (23). That is, ‘controlled’ WC (*n* = 76) for men and women was less than 85 cm and 90 cm, respectively, and ‘controlled’ IAF (*n* = 96) was less than 100 cm^2^ for both men and women (23). All results were expressed after adjustment was made for three confounders: age, gender and number of anti-diabetic medications being used among the nine classes presented in Table 1 (insulin, sulfonylureas (SU), glinides, thiazolidinedione (TZD), biguanide (BG), alpha-glucosidase inhibitors (α-GI), dipeptidyl peptidase-4 inhibitors (DPP-4I), sodium-glucose co-transporter 2 inhibitors (SGLT2I), glucagon-like peptide 1 receptor agonists (GLP-1RA)) because the drugs are known to affect not only bodyweight but also HbA1c level, depending on the type of medication. To evaluate model fitness, we referred to the Somers’ D and c-statistics. *P*-values of < 0.05 were considered to indicate statistical significance. Statistical analyses were performed using JMP software version 13.2 (Windows, SAS Institute). No statistical sample size calculations were conducted in advance because of the retrospective nature of this study. However, each of the sample sizes of 64, 81 and 633 for HbA1c; 17, 48 and 609 for BMI; 13, 40 and 423 for WC; and 15, 47 and 390 for VFA, respectively, in the HS, HL and LS groups would have 80% post-hoc power to detect differences when compared with the LL group using a two-group *t*-test with a two-sided significance level of *P* < 0.05 if the calculations were to be carried out.

## Results

### Clinical Characteristics of Study Participants

Among the 270 study participants, mean age, HbA1c, BMI, WC and IAF for all participants at the beginning of the study were 63.8 (11.2) years old, 7.31 (1.27%), 25.8 (4.4) kg/m^2^, 93.9 (10.9) cm and 123 (50) cm^2^, respectively. Mean hand strength, GS and ST were 28.9 (9.8) kg, 0.43 (0.12), 410 (215) min/day, respectively. Table 1 indicates the clinical characteristics of the participants categorised into the four groups (LL, LS, HL and HS). Participants in the HL and HS groups had significantly lower values for HbA1c than participants in the LL group. In addition, participants in the HS group had significantly lower values for each of BMI, WC and IAF than participants in the LL, LS and HL groups. No differences were observed regarding gender distribution, systolic and diastolic blood pressure, liver and renal functions, cholesterol levels, and treatments for diabetes among the four groups.

### The Differences in HbA1c, BMI, WC, IAF and GS Based on ST

Mean BMI, WC, IAF and GS in this study among participants in the category with short ST (LS and HS) were lower than those among participants in the category with long ST (LL and HL). The differences were statistically significant (*P* = 0.005, < 0.001, < 0.001 and < 0.001, respectively) based on ANCOVA analyses. The differences in BMI, WC and IAF were statistically significant after further adjustment was made for hand strength. However, the mean HbA1c in this study did not differ between participants with short and long ST. In addition, among participants in the category with long ST, BMI, WC and IAF in the low GS group were significantly higher than in high GS (*P* < 0.001, each), as well as among participants in the category with short ST (*P* < 0.001, each).

### The Differences in HbA1c, BMI, WC, IAF and ST Based on GS

Mean HbA1c, BMI, WC, IAF and ST in this study among participants in the category with high GS (HL and HS) were all lower than those among participants in the category with low GS (LL and LS). The differences were statistically significant (*P* = 0.003, < 0.001, < 0.001, < 0.001 and 0.003, respectively), based on ANCOVA analyses. The differences in HbA1c, BMI, WC and IAF were statistically significant after further adjustment was made for ST. In addition, among participants in the category with high GS, BMI, WC and IAF in the long ST group were significantly higher than in short ST (*P* = 0.026, 0.002 and 0.008, respectively), although not among participants in the category with low GS.

### The Impact of GS and ST on Association with HbA1c, BMI, WC and IAF

First, the study examined the impact of GS and ST on current glycaemic control, by categorising the participants into four groups according to GS and ST (Table 2). As a result, after adjustment was made for the above-mentioned three confounders, the participants in the HS group were found to have a higher OR of 2.01 (95% CI: 1.003, 4.03; *P* = 0.049) for controlled HbA1c, compared with that in the LL group. In contrast, the participants in the LS and HL groups had ORs of 0.62 (95% CI: 0.29, 1.33; *P* = 0.221) and ORs of 1.02 (95% CI: 0.48, 2.16; *P* = 0.956) for controlled HbA1c. However, the ORs were significantly increased with a positive trend in order from LL, LS, HL to HS (*P* = 0.025 for trend). Next, we examined the impact of GS and ST on current BMI and body composition. As a result, the participants in the HL and HS groups had significantly higher ORs for controlled BMI, WC and IAF compared with those in the LL group, as follows: controlled BMI, for HL, 6.05 (95% CI: 2.58, 14.18; *P* < 0.001) and for HS, 11.86 (95% CI: 4.90, 28.73; *P* < 0.001); controlled WC, 3.76 (95% CI: 1.36, 10.43; *P* = 0.011) and 9.78 (95% CI: 3.86, 24.77; *P* < 0.001), respectively; and controlled IAF, 2.46 (95% CI: 1.02, 5.96; *P* = 0.046) and 9.78 (95% CI: 3.86, 24.77; *P* < 0.001), respectively. In addition, the ORs were significantly increased with a positive trend in order from LL, LS, HL to HS in all three analyses (*P* < 0.001 for trend, each).

## Discussion

This cross-sectional study’s results indicated that GS was associated with HbA1c, bodyweight, and body composition among patients with T2DM. Sedentary behaviour affected body composition especially among T2DM patients with high GS. To the best of our knowledge, this was the first study to analyse the impact of hand strength in consideration of sedentary behaviour on present glycaemic control, bodyweight and body composition in Japanese patients with T2DM.

This study found that the values for BMI, WC and IAF were significantly lower for high GS compared with low GS, whereas association between hand strength and WC (24) and BMI (25) was previously reported. Moreover, this study found that participants in the high GS group had well controlled BMI, WC and IAF, compared with those in the low GS and long ST group, regardless of ST. Such relationships observed in those groups primarily suggest that bodyweight and body composition might readily be affected by a patient’s own muscular strength, because skeletal muscle metabolism is assumed to play a role in resting energy expenditure (26). Accordingly, the current study suggests that to prevent overweight or accumulation of visceral fat and the exacerbation of diabetes symptoms, improvement in muscular strength could be an important element.

High GS also had low HbA1c compared to low GS in this study, as well as in another study of older patients with T2DM (27). In addition, high OR for glycaemic control was found most commonly among high GS participants with short ST compared to those with long ST. The benefit of increasing non-sedentary activities for glycaemic control appeared more significantly among patients with high GS than in those with low GS. However, HbA1c values could have been the result of use of diabetes medications prescribed by the attending physician to target HbA1c level. To clarify the association between glycaemic control and GS or ST in more detail, further study is necessary with a larger number of diabetes patients.

According to a self-recorded questionnaire for T2DM patients conducted in Japan (28), the implementation rate of exercise therapy was only about 50% in diabetes patients, with 30% of such patients never receiving instruction regarding exercise, contrary to only 9.9% of patients never receiving advice about nutrition. The questionnaire results might indicate that instruction about exercise therapy for T2DM is difficult to convey to, and be understood by, patients. Taking such circumstances into consideration, the results of this study might permit a simple but intriguing approach in which patients with high hand strength but long ST would be encouraged to simply interrupt prolonged sitting to promote increased glucose metabolism (29) as well as better bodyweight and body composition. In contrast, among patients with weakened muscular strength, increasing the time in resistance training might be a reasonable approach independent of ST, whereas maintaining short durations of ST could be a critical surrogate treatment strategy for disease management in patients with T2DM (13).

Interestingly, the ORs were significantly increased with a positive trend in order from LL, LS, HL to HS in all three analyses in the study. To take into consideration the results between LS and HS, hand strength might be more important for the treatment of T2DM, not only for HbA1c but also for body composition, than sedentary behaviour. In other words, the effect of muscular strength might be considerable and compensate for weak muscular strength such as weak hand through the interruption of sedentary behaviour. Further prospective study is needed.

The present study has several limitations. First, it was a cross-sectional, retrospective, single-centred study with a limited participant population, in which systematic bias might have remained. However, a post-hoc power calculation has previously been deemed not appropriate (30). The generalisability of the results resulting from this study is therefore limited. Second, diabetes medications could have modified patient bodyweight. Assessing the effect independently of those medications was difficult due to the study design, even though statistical adjustment was made for medication effects. Third, habits and comorbidity factors such as smoking, cognitive function, frailty and daily activities were not considered. Fourth, ST information was obtained on the basis of the IPAQ short form without use of any monitoring devices. Lastly, IAF was methodologically assessed only by BIA and not by CT. Further prospective study is required to clarify the precise impact of sedentary behaviour on physical activity and management of T2DM.

## Conclusion

In conclusion, the results of this study suggest that instruction regarding physical activity for treatment of T2DM should be adapted based on patient muscular strength as measured by hand strength and sedentary behaviour estimated by ST duration. Patients’ sedentary behaviour might modestly impact muscular strength and control of HbA1c and body composition.

## Figures and Tables

**Table 1:** Clinical characteristics in each group based on sedentary time and hand strength among patients with T2DM

	Low GS		High GS	

	Long ST (LL)	Short ST (LS)	Long ST (HL)	Short ST (HS)
Male/Female (*n*)	49/28	33/25	39/19	43/34
Age (years old)	63.9 (11.8)	65.9 (10.9)	60.3 (9.6)†	64.7 (11.5)
BMI (kg/m^2^)	27.7 (4.9)	27.0 (4.3)	25.2 ± 3.5*	23.4 ± 3.0*†‡
HbA1c (%)	7.65 (1.66)	7.45 (1.06)	7.09 (1.15)*	7.02 (0.93)*
SBP (mmHg)	138 (19)	133 (15)	131 (17)	131 (14)
DBP (mmHg)	78 (14)	78 (12)	80 (12)	76 (12)
AST (U/L)	24.3 (9.1)	22.7 (9.1)	23.0 (8.6)	25.4 (26.7)
ALT (U/L)	25.5 (14.5)	22.7 (12.2)	25.8 (14.9)	24.6 (24.3)
eGFR (mL/min/1.73m^2^)	73.2 (24.5)	76.2 (19.7)	71.8 (17.1)	78.2 (20.1)
TCH (mg/dL)	179 (35)	193 (36)	193 (40)	182 (33)
TG (mg/dL)	169 (98)	119 (65)	187 (206)†	120 (75)‡
HDLC (mg/dL)	51 (14)	57 (16)	51 (11)	54 (13)
LDLC (mg/dL)	99 (30)	112 (32)	106 (26)	105 (30)
WC (cm)	99.3 (10.9)	97.2 (11.4)	92.4 (8.0)*†	87.0 (8.4)*†‡
VFA (cm^2^)	146 (48)	136 (51)	117 (39)*	95 (43)*†‡
Sedentary time (min/day)	595 (184)	248 (76)	557 (159)	239 (78)
Hand strength (kg)	25.0 (8.4)	25.6 (8.7)	34.7 (10.3)*†	31.0 (8.9)*†‡
GS	0.34 (0.09)	0.36 (0.08)	0.50 (0.09)*†	0.50 (0.09)*†
Treatment for diabetes (*n*)				
Insulin/GLP-1RA	12/10	6/5	7/5	9/5
BG/α-GI/DPP-4I	39/5/31	32/2/24	21/2/13	28/2/33
TZD/SGLT2I	3/33	2/24	0/15	3/22
SU/Glinides	9/1	8/4	4/3	10/5

Note: mean (SD);

* *P* < 0.05 compared to category of low GS and long ST;

† *P* < 0.05 compared to category of low GS and short ST, and

‡ *P* < 0.05 compared to category of high GS and long ST after adjustment for confounders, respectively

**Table 2:** Adjusted odds ratio for various clinical parameters such as HbA1c, BMI, WC and IAF among participants who were categorised into the four groups

	Events/population	OR	95% CI	*P*-value
Controlled HbA1c				
Low GS/long ST (LL)	33/77	1.00		
Low GS/short ST (LS)	20/58	0.623	0.291, 1.330	0.221
High GS/long ST (HL)	29/58	1.021	0.483, 2.159	0.956
High GS/short ST (HS)	48/77	2.010	1.003, 4.026	0.049
P for trend		1.289		0.025
Controlled BMI				
Low GS/long ST (LL)	30/77	1.00		
Low GS/short ST (LS)	28/58	1.455	0.666, 3.175	0.347
High GS/long ST (HL)	40/58	6.050	2.581, 14.183	< 0.001
High GS/short ST (HS)	63/77	11.861	4.897, 28.729	< 0.001
P for trend		2.370		< 0.001
Controlled WC				
Low GS/long ST (LL)	9/77	1.00		
Low GS/short ST (LS)	13/58	2.002	0.729, 5.499	0.178
High GS/long ST (HL)	15/58	3.759	1.355, 10.430	0.011
High GS/short ST (HS)	39/77	9.779	3.861, 24.767	< 0.001
P for trend		2.148		< 0.001
Controlled VFA				
Low GS/long ST (LL)	15/77	1.00		
Low GS/short ST (LS)	14/58	1.132	0.463, 2.767	0.786
High GS/long ST (HL)	18/58	2.460	1.016, 5.956	0.046
High GS/short ST (HS)	49/77	8.881	3.900, 20.221	< 0.001
P for trend		2.146		< 0.001

Note: mean (SD);

* *P* < 0.05 compared to category of low GS and long ST;

† *P* < 0.05 compared to category of low GS and short ST, and

‡ *P* < 0.05 compared to category of high GS and long ST after adjustment for confounders, respectively
